# Erkennung transparenter Objekte für die Laborautomatisierung

**DOI:** 10.1007/s00502-023-01158-w

**Published:** 2023-09-12

**Authors:** Markus Vincze, Jean-Baptiste Weibel, Stefan Thalhammer, Hrishikesh Gupta, Philipp Ausserlechner

**Affiliations:** Automatisierungs- und Regelungstechnik Institut, TU Wien, Gusshausstr. 27/376, 1040 Wien, Österreich

**Keywords:** Bildverarbeitung, Transparente Objekte, Roboter, Objekterkennung, Posebestimmung, Robotics, Computer vision, Detection, Transparent objects, Pose estimation, Grasping

## Abstract

Während matte Objekte visuell gut erkannt und mit Robotern gegriffen werden können, stellen transparente Objekte neue Herausforderungen dar. So liefern moderne Farb- und Tiefenbildkameras (RGB-D) keine korrekten Tiefendaten, sondern verzerrte Abbildungen des Hintergrunds. Wir zeigen in diesem Beitrag, welche Methoden geeignet sind, um nur in Farbbildern transparente Objekte zu erkennen und deren Pose zu bestimmen. Mittels eines Robotersystems werden Ansichten des Zielobjekts generiert und annotiert, um Methoden anzulernen und um Daten für die Evaluierung zu erhalten. Wir zeigen auch, dass mittels einer verbesserten Methode zum Einpassen der 3D-Pose eine deutliche Verbesserung der Genauigkeit der Lageschätzung erreicht wird. Dadurch können falsche Erkennungen aussortiert werden und für richtige Erkennungen wird die Genauigkeit der Poseschätzung verbessert. Somit gelingt es, mit einem Roboter transparente Objekte zu greifen.

## Abstract (en)

While matte objects can be visually recognized well and grasped with robots, transparent objects pose new challenges. Modern color and depth cameras (RGB-D) do not deliver correct depth data but distorted images of the background. In this paper, we show which methods are suitable to detect transparent objects in color images only and to determine their pose. Using a robotic system, views of the targeted object are generated and annotated to learn methods and to obtain data for evaluation. We also show that by using an improved method for fitting the 3D pose, a significant improvement in the accuracy of pose estimation is achieved. Thus, false detections can be eliminated and for correct detections the accuracy of pose estimation is improved. This makes it possible to grasp transparent objects with a robot.

## Einleitung

In der Laborautomation dominieren auch heute noch manuelle Verfahren bei der Montage und Prüfung von Produkten für pharmazeutische und medizinische Anwendungen [1]. Der Grund dafür ist, dass die Vorschriften für eine sichere Ausführung jedes Prozessschrittes eine systematische Überprüfung der korrekten Durchführung der Montageaufgabe verlangen. Man spricht von der Erstellung eines *Prüfprotokolls* oder auch Audit Trail genannt [2]. Eine besondere Herausforderung in diesem Bereich ist der Umgang mit sterilen Medizinprodukten. In diesem Bereich wäre eine Automatisierung wünschenswert, denn Menschen können nur in Schutzkleidung in die sterile Umgebung gebracht werden. Eine Lösung ist daher die Laborautomatisierung mittels geeigneter Methoden der Robotik und künstlichen Intelligenz (KI).

Derzeit erfolgt die Erstellung des Prüfprotokolls durch den Menschen. Dabei wird in der Montageliste jeder Schritt nach der Durchführung als erfolgreich bestätigt. Diese manuelle Nachverfolgbarkeit soll nun durch eine automatisierte Verifikation durch das Robotersystem ersetzt werden.

Erste Ansätze zeigen, dass für einfache Montageaufgaben mit vordefinierten Maschinen diese Arbeitsschritte erstellt werden können [3] oder die Korrektheit der Abfolge überprüft werden kann [4]. Für spezifische Schritte wie das Schließen von Schnappverschlüssen gibt es erste Möglichkeiten einer Verifikation [5]. Weitere Ansätze versuchen Bildverarbeitung für die Verifikation der Pose des Objektes einzusetzen [6–8]. Derzeit ist es jedoch nicht möglich, ein Prüfprotokoll für die einzelnen Schritte einer Montage mit Robotern zu erstellen.

**Figure 1:**
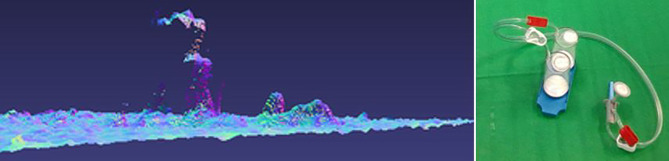

In diesem Beitrag berichten wir über Lösungen um in Bilddaten die Erkennung und Poseschätzung von bekannten Objekten zu überprüfen und zu verifizieren. Insbesondere wenden wir die Methoden darauf an, die Objekte eines Sterilitätsprüfsets zu erkennen, siehe Abb. 1 rechts. Dabei gilt es besonders die Herausforderung, welche mit der Transparenz der Materialen einhergeht, zu lösen. Die Geometrie der Objekte muss robust erkannt werden, obwohl die Sensorik keine Objekttextur, sondern eine verzerrte Abbildung des Hintergrunds liefert.

Für die Erkenung und Posebestimmung von transparenten Objekten präsentieren wir hier Adaptionen von bestehenden und neue Lösungen, die insbesondere folgende Aspekte umfassen:

1. eine Methode um transparente Objekte zu erkennen und deren Pose zu schätzen,
2. eine Methode zur Verifizierung der Objektklasse als auch der exakten Pose des Objektes,
3. eine Erweiterung des Ansatzes um transparente Schläuche und deren Verlauf zu bestimmen, und
4. eine Lösung um mit einem Roboter Modelle der Objekte als auch große Datenmenge (mehrere 1000 Bilder) für das Lernen und Evaluieren der Methoden, in weniger als einem Arbeitstag, aufzunehmen.

Der Beitrag startet mit einer Analyse der Probleme bei der Erkennung von transparenten Objekten, Kap. 2, präsentiert dann die Methoden und Ergebnisse in Kap. 3 und die Lösung zur Erfassung von Modellen und Daten fürs Lernen und Evaluieren in Kap. 4. Als Abschluss folgt ein Beispiel um mit einem Serviceroboter ein transparentes Objekt zu greifen in Kap. 5.

## Offene Probleme bei der Erkennung von transparenten Objekten

Die letzten Jahre zeigten große Fortschritte bei Methoden zur Erkennung von Objekten mit Farb- und Tiefendaten mit RGB‑D Kameras wie der Kinect oder der RealSense. Während Farbe gut ist um Objekte zu erkennen, helfen Tiefendaten um die Pose von Objekten genauer zu bestimmen [7]. Für transparente und reflektierende Objekte ergeben sich jedoch Probleme bei der Bildaufnahme mit Tiefenbildkameras.

Das eine Problem dieser Tiefenkameras ist, dass sie aktiv Licht aussenden, meist im infraroten Spektrum, und daher transparente und reflektierende Materialien nicht erfasst werden können [9]. Abb. 1 links zeigt ein Beispiel mit den Kanistern des Sterilitätsprüfsets. Man sieht in der Ansicht von der Seite, dass nur Artefakte im Tiefenbild auftauchen. Daher müssen Methoden mit Farbbilddaten auskommen um transparente Objekte zu erkennen.

Das zweite Problem hängt mit dem Aufkommen von Methoden des Deep Learning (Neuronalen Netzwerken mit vielen Ebenen) zusammen. Nach diesem Ansatz arbeiten derzeit fast alle Methoden auf Basis von Convolutional Neural Netzwerks (CNN) [10]. Deep Learning benötigt große Mengen an Trainingsdaten, und damit einhergehend auch Annotationen der Bilder (Objektklasse, Position im Bild und Pose im $3D$-Raum).

**Figure 2:**
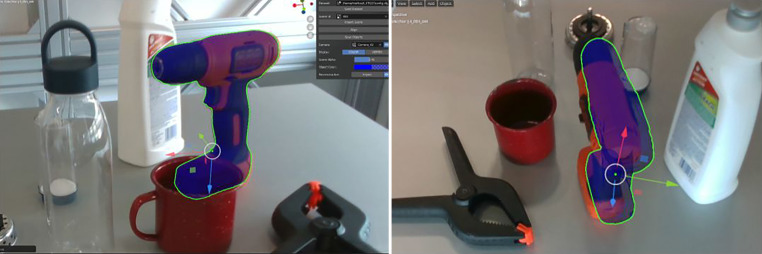

Die Schwierigkeit dabei ist die dreidimensionale Pose aus den zweidimensionalen Bildern richtig zu schätzen. Abb. 2 veranschaulicht das Problem. Da ein Farbbild keine Tiefeninformation liefert, ist die Poseschätzung in der Tiefe schlecht. Obwohl die Pose anhand der Kontur des Gegenstandes sehr genau aussieht, ist die Distanzschätzung im Tiefenbild um mehr als einen Zentimeter falsch. Als Folge davon sind viele Referenzdaten derzeitiger Datensets nicht sehr genau und daher auch die Genauigkeit der Aussagen von solchen Datensets beschränkt. Wir zeigen im nächsten Abschnitt, wie eine Poseschätzung von transparenten Objekten in Farbbildern möglich ist, und danach in Abschn. 4, wie man die Genauigkeit von Datensets verbessern kann.

## Transparente Objekte: Erkennung und Bestimmung der Lage und Orientierung

Im Zuge unserer Forschungen entwickeln wir Methoden um direkt aus Farbbildern die Pose von Objekten zu bestimmen und daraus Greifpunkte für die Manipulation mit dem Roboter abzuleiten [11, 12]. Dabei zeigt sich, dass Poseverifizierung in der Simulation, basierend auf physikalischer Plausibilität, vergleichbare bis bessere Genauigkeiten erzielt, wie Methoden zur Posenverifizierung welche echte Objektobservationen verwenden (Kap. 3.2) [13]. So wie alle Methoden um Objekte zu erkennen, ist das Ergebnis eine Hypothese, dass sich an dieser Stelle im Bild ein Objekt einer bestimmten Klasse befindet. Ein Konfidenzmaß gibt an, wie sicher die Methode ist, dass es sich um dieses Objekt handelt. Obwohl die Erkennungsmethoden laufend besser werden, sind diese Hypothesen unsicher. Um diese Unsicherheit zu minimieren wird ein zusätzlichen Schritt, zur Verifizierung der Objektpose angewandt [7]. Dieser Ansatz, Hypothesenbildung und Verifizierung, verbessert die Ergebnisse in vielen Anwendungen deutlich (Kap. 3.2).

### Hypothesenbildung: Objekterkennung und Posebestimmung

COPE (Constant Runtime Object Pose Estimation – Konstante Laufzeit für die Posebestimmug von Objekten) [14] basiert auf der Idee eine geometrische Repräsentation von vielen Objekten zu lernen und daraus direkt die 6D Pose (Lage und Orientierung) zu bestimmen. Dies erfolgt gleichzeitig für alle im Bild gefundenen Objekte, so dass wir von konstanter Laufzeit sprechen können, da die inhärente End-zu-End-Trainierbarkeit von COPE die Anforderung überwindet, einzelne Objektinstanzen separat zu verarbeiten.

COPE baut auf dem Erfolg von effizienten neueren Objekterkennungsansätzen auf. Das RGB-Eingabebild wird zunächst mit einem CNN-Backbone verarbeitet und dann werden mithilfe einer Bildpyramide über mehrere Größenoktaven Merkmale berechnet, um die Zwischenrepräsentation des Objekts zu schätzen. Durch die Berechnung der gegenseitigen Überschneidungen werden die Posenhypothesen in verschiedene Instanzen zusammengezogen. Dies führt nur zu einer vernachlässigbaren Laufzeiterhöhung in Bezug auf die Anzahl der Objektinstanzen. Die Ergebnisse auf mehreren anspruchsvollen Standarddatensätzen zeigen, dass die Leistung der Posenschätzungen den Stand-der-Technik Ansätzen mit einem Modell überlegen ist, obwohl COPE mehr als 35 Mal schneller ist. Einzelheiten zu dieser Methode befinden sich in [14].

**Figure 3:**
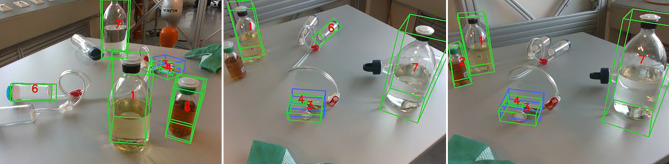

Wir haben die COPE Methode speziell an transparenten Objekten trainiert. Das Ziel ist die Klassifizierung und Schätzung der Posen aller Objektinstanzen in einem einzigen RGB-Eingabebild. Es wird davon ausgegangen, dass die Objektmodelle im Voraus bekannt sind, aber keine zusätzlichen Informationen über die Testszene erforderlich sind. Wir definieren die Eckpunkte des kleinsten Quaders als geometrische Korrespondenzen, der das jeweilige Objektnetz in seinem Koordinatensystem umschließt. COPE erzeugt dann die Menge der im Bild sichtbaren Objektinstanzen, parametrisiert mit Objekttyp und 6D-Pose. Eine Evaluierung der COPE-Methode mit den Objekten aus dem Sterilitätsprüfset zeigt, dass wir für 70 Prozent der Objekte gute Posen für das Greifen der Objekte erhalten. Beispielhafte Ergebnisse sind in Abb. 3 dargestellt.

### Verifizierung: Bestätigung und Verfeinerung der Objektpose

COPE als auch andere Methoden zur Objekterkennung liefern Hypothesen, in diesem Fall die Klasse des erkannten Objektes und die Pose des Objektes für die Manipulation mit dem Roboter. Der Schritt einer Verifizierung der Pose erfolgt durch eine iterative Verfeinerung der Pose mittels einer Simulation in zwei Schritten: (1) zuerst wird die physische Plausibilität geprüft und (2) danach mittels einer Rückprojektion die simulierte Welt mit der beobachteten in Übereinstimmung gebracht wird.

**Figure 4:**
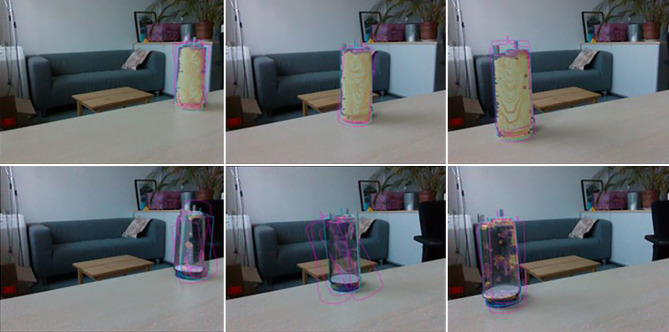

In Schritt (1) nutzen wir eine Physiksimulation, um die visuelle Ausrichtung als auch die physikalische Plausibilität der Poseschätzungen zu verbessern. Wie in Abb. 4 hervorgehoben, kann dieser Ansatz eine Reihe von anfänglichen Posehypothesen (magenta) verfeinern und die visuell am besten ausgerichtete Pose für jedes Objekt (cyan) unter Verwendung der unten beschriebenen Rückprojektion und deren Bewertung bestimmen. Dabei kommt auch die Geometrie der Objektumgebung zu Hilfe und bestimmt eine plausiblere Pose des Objektes sowie eine bessere visuelle Ausrichtung. Die Beispiele in der Abbildung zeigen, dass diese Methode besonders für transparente Objekte deutliche Verbesserungen erzielt.

Im ersten Schritt erfolgt die Verbesserung der Objektpose durch die physikalische Simulation einer plausiblen Pose (Abb. 4 obere Reihe). Das Objekt wird in Bezug auf die detektierte Ebene in einer stabilen Pose ausgerichtet und so verschoben, dass es genau in Kontakt mit der Ebene steht. Die Verschiebung in der Ebene wird zusätzlich durch den auf die Ebene projizierten Schwerpunkt der Segmentierungsmaske aus dem Farbbild angenähert. Dabei nutzen wir die Hypothese der Objektpose aus COPE (oder beliebigen anderen Methoden, die eine Objekthypothese liefern) oder weiteres Vorwissen über die ungefähre Objektposition wie Daten aus vorherigen Bildern, die über die Simulation im digitalen Zwilling abrufbar sind.

Im zweiten Schritt wird die bereits verbesserte Pose durch eine Rückprojektion (inverse rendering) verfeinert. Dabei benützen wir die Übereinstimmung der rückprojizierten Maske (oder den Umriss oder Kontur) des Objektes aus der Simulation um eine differentielle Änderung abzuleiten. Die differenzierbare Rückprojektion liefert den Verlustgradienten in Bezug auf die Objektposition. Diese wird für die Optimierung der Maskeneinpassung herangezogen, die iterativ eine genaue Ausrichtung der rückprojizierten mit der beobachteten Objektmasken durchführt.

Auch in diesem zweiten Schritt nutzen wir die Geometrie der Szene und beschränken die Optimierung auf die Rotation in der Ebene und die Translation in der Ebene. Die resultierende Pose bleibt daher in Bezug auf die erkannte Ebene plausibel. Die endgültigen Posen für den transparenten Kanister sind in Abb. 4 (unten) dargestellt. Dieses Beispiel verdeutlicht die Generalisierung des Ansatzes auf matte, wie auch auf transparente Objekte, da er nur von einer Segmentierungsmaske als Ziel und einem 3D-Netz für die differenzierbare Darstellung abhängt.

### Transparente Schläuche

Während die oben genannten Ansätze zum Erkennen und Erfassen starrer, transparenter Objekte führen, präsentieren viele Anwendungen auch transparente Schläuche wie sie häufig im medizinischen oder pharmazeutischen Bereich zum Einsatz kommen. Die Schläuche sind verformbar aber besitzen Eigenschaften, die zur Erkennung genutzt werden können. Beispiele sind die bekannten Anfangs- und Endpunkt der Schläuche, die mit den roten und weißen Clips in der Nähe der Nadel und durch den transparenten Kanister definiert sind. Auch Teile des Kanisters oder der Nadel könnten als Endstücke der Schläuche erkannt werden.

**Figure 5:**
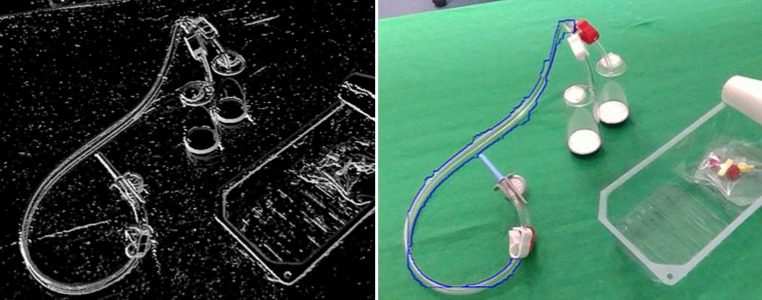

Da die Schläuche transparent sind, liefern auch hier die Tiefenbilder keine nützlichen Daten. Abb. 1 zeigt ein Beispiel, bei dem Teile des Kanisters, aber nur sehr wenig von den Schläuchen zu sehen sind. Im Farbbild jedoch ist der Schlauch vollständig sichtbar. Die Lösung muss daher die Farbbilddaten verwenden. Wie die Bildanalysen zeigen, sind Kanten weiterhin gut sichtbar. Da die Schläuche eine bestimmte Dicke aufweisen, gilt es parallele Kantenzüge zu finden. Da die konkreten Schläuche in der medizinischen Anwendung doppelte Schläuche sind, kommt es je nach Blickrichtung auch zu mehr als zwei Kanten. Abb. 5 zeigt ein Beispielbild der Kanten.

**Figure 6:**
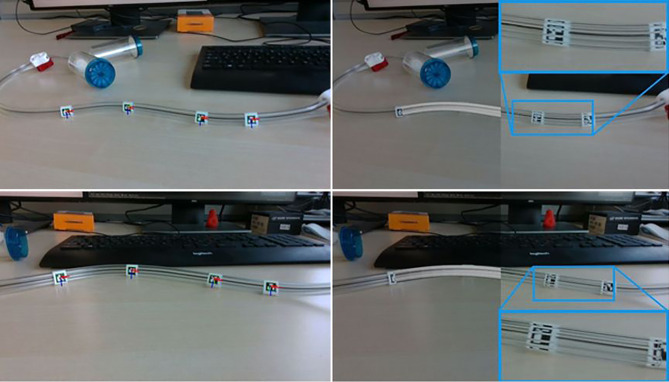

Um Schläuche zu erkennen, wird auch die bekannte Steifigkeit ausgenutzt. Es folgt daraus, dass die Änderung der Krümmung der Schläuche begrenzt ist und sich auch nur langsam ändern kann. Um die Krümmung zu erhalten, wird die zweite Ableitung aus den Richtungen der Kantenzüge berechnet. Die resultierenden Orientierungen werden in Bereiche mit ähnlicher Krümmungsänderung sortiert und mit angrenzenden Bereichen verbunden. Schläuche werden somit als Kurvenmodelle in die Pixel des Farbbildes eingepasst. Dieses Verfahren liefert gute Ergebnisse für die Erkennung von Schläuchen und deren Verlauf. Ein Beispiel ist in Abb. 6 zu sehen.

**Figure 7:**
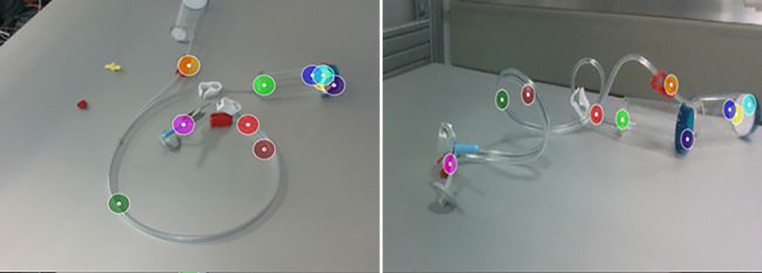

Während diese Methode gute Ergebnisse zeigt, können auch bestimmte Objekte oder Teile entlang des Schlauches benützt werden, um die Erkennung zu verbessern. Diese Objekte sind kleiner als die Kanister, erlauben aber eine semantische Zuordnung entlang des Schlauches und damit auch eine Überprüfung, da die Anordnung der Teile entlang des Schlauches fix vorgegeben ist. Für diese Aufgabe verwenden wir selbstüberwachte Lernmethoden (Emerging Properties in Self-Supervised Vision Transformers [15, 16]). Wir modifizieren die Inferenzpipeline für die Poseschätzung um unsere Ergebnisse der Poseverifizierung verwenden zu können. Die bisherigen Ergebnisse zeigen, dass wir gute semantische Korrespondenzen auf dem Schlauch mit den Klammern und Nadeln erhalten, wie Abb. 7 zeigt.

## Datasets und Evaluierung

Um den Fortschritt bei der Erkennung von transparenten Gegenständen zu evaluieren, werden geeignete Daten benötigt. Erste Datensätze, die für die Erkennung von transparenten Objekten geeignet sind, wurden in Arbeiten publiziert wie ClearGrasp [17], KeyPose [18] und TODD [19]. Diese Datensätze enthalten meist leere transparente Gefäße, mit Ausnahme des Toronto Transparent Object Depth (TODD)-Datensatzes, der auch mit Flüssigkeit gefüllte Objekte enthält, allerdings nur für sechs verschiedene Objekte. Diese Datensätze liefern auch die Referenz für die Position und Lage der Objekte in Form von Masken und Objektposen, die für das Lernen und die Bewertung notwendig sind.

Während diese Datensätze eine erste Grundlage darstellen, müssen wir bewerten, wie Methoden insbesondere für die relevanten Objekte der Anwendung abschneiden. Aus diesem Grund ist es notwendig ein Datenset für die Zielobjekte zu erstellen. Da dies auch für viele andere Anwendungen gilt, stellen wir hier ein effizientes Verfahren vor, wie exakte Objektdaten und deren Posen erlangt werden können.

### Erstellen von Daten für neue Anwendungen

Die Herausforderungen, um Daten zum Lernen und Testen von bekannten und neuen Methoden für eine spezielle Anwendung zu erhalten, sind (1) die rasche Aufnahme von genügend Daten und (2) eine hochgenaue Annotation der Objektpose. Die Erstellung der Daten erfolgt automatisiert mittels eines Roboters, der über die Szene schwenkt und dabei 104 Bilder aus verschiedenen Blickwinkeln aufnimmt. Die Annotation erfolgt über zwei orthogonale Ansichten, sodass nur einmal für alle 104 Aufnahmen einer Szene die genaue Objektpose bestimmt werden muss.

Der TraceBot^1^ Datensatz umfasst RGB-D-Bilder von transparenten Objekten von 10 Szenen. Zusätzlich zu den RGB- und Tiefenbildern enthalten die Daten auch Infrarotbilder und die zugehörige Kameraposition. Beispiele für die in diesem Datensatz enthaltenen Informationen sind in Abb. 11 dargestellt. Da jede Szene aus 104 Ansichten aufgenommen wurde, kann der Datensatz auch zur Bewertung von Rekonstruktionsmethoden mit mehreren Ansichten verwendet werden.

**Figure 8:**
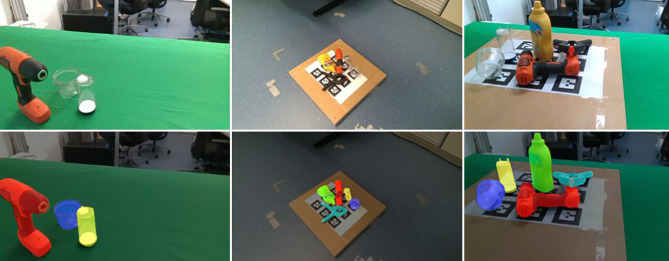

Der TraceBot Datensatz enthält zwei verschiedene Arten von transparenten Objekten: gewöhnliche transparente Haushaltsgegenstände sowie transparente Behälter und Flaschen, die für verschiedene medizinische Anwendungen verwendet werden und insbesondere aus dem Anwendungsfall – dem Sterilitätsset – stammen. Die Auswahl umfasst Objekte unterschiedlicher Komplexität, die von einfachen symmetrischen und flachen Objekten bis hin zu Objekten mit mehr und feineren Details sowie mit Inhalt gefüllten Gefäßen reicht. Abb. 8 gibt einen Überblick über die typischen Szenen in unserem Datensatz.

### Rasche Aufnahme von genügend Daten

**Figure 9:**
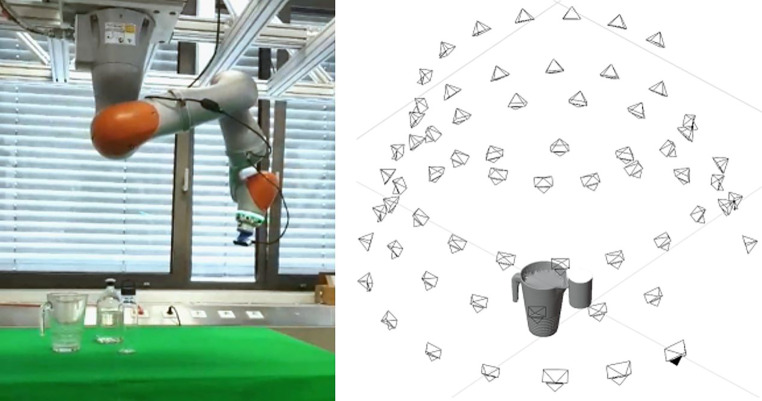

Der Aufbau für die Datenerfassung ist in Abb. 9 dargestellt: Er besteht aus einem 7 DoF Kuka Robotermanipulator mit einer RGB-D-Kamera, die an seinem Endeffektor befestigt ist. Es wurde eine RealSense D435-Kamera ausgewählt, da sie sowohl die Erfassung von RGB-D-Bildern als auch von Infrarotbildern ermöglicht. Die Auge-in-Hand Konfiguration wird mit Hilfe von Referenzmarkern kalibriert. Der Vorteil dieses Aufbaus ist, dass schnell viele Ansichten von einer Szene erstellt werden können aber mit der Annotation einer Sicht alle Ansichten eine genaue Referenzpose erhalten [20]. Der letzte Schritt ist besonders wichtig, da die Erfassung der Objektpositionen sehr zeitaufwändig ist. Indem wir die bekannten Kamerapositionen ausnutzen, übertragen wir die Objektposen automatisch in andere Ansichten, indem wir den in [20] vorgestellten Ansatz mit unseren Werkzeugen verwenden.

**Figure 10:**
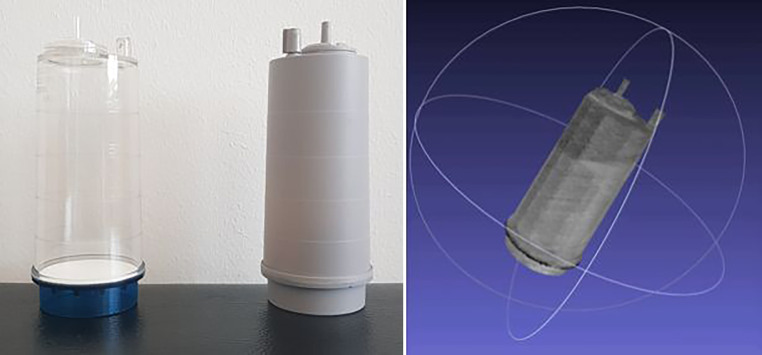

Wie oben erläutert können derzeitige Methoden direkt aus Modelldaten gelernt werden. Wenn CAD Modelle vorhanden sind, hilft das Aufbringen von Texturen. Wenn keine Modelle vorhanden sind, können mit dem Roboter, wie in der obigen Abbildung gezeigt, auch sehr rasch die Modelle aufgebaut werden. Für transparente Objekte hilft es einmal ein lackiertes Objektes zu verwenden, um eine exakte Referenzgeometrie abzuleiten. Dabei verwenden wir NeRF (Neural Radial Field) Modelle [20]. Abb. 10 zeigt ein Beispiel.

Das Ergebnis ist ein Datensatz mit Szenen verschiedener Komplexität von einzeln-stehenden bis zu dicht-stehenden Objekten mit jeweils 104 Ansichten. Abb. 8 zeigt Beispiele.

### Annotation der Objektpose

**Figure 11:**
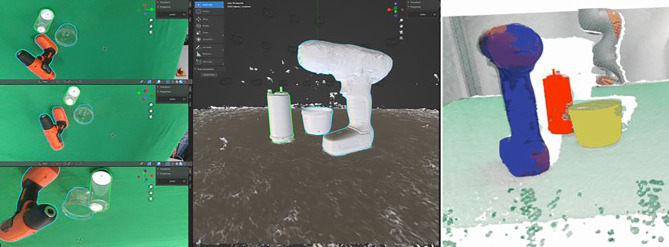

Wir haben die Benutzeroberfläche für unser Annotationstool als Blender-Add-on erstellt [20]. Die von Blender angebotenen Widgets zum Verschieben und Drehen werden verwendet, um Objekte zu platzieren und auszurichten. Der Benutzer kann schnell navigieren und die importierten Kameraansichten durchsuchen und hat ein permanentes visuelles Feedback von der Rückprojektion des 3D-Modells. Der Benutzer kann auch mit transparenten 3D-Modellen arbeiten, um Anpassungen leichter sichtbar zu machen, indem z. B. nur die Silhouette des Objekts verwendet wird. Das 3D-DAT Tool ist in ROS integriert und lässt sich einfach bedienen. Es kann für die Aufnahme von Bild‑, Punktwolken- und Pose-Themen konfiguriert werden. Weitere Details finden sich in [20]. Abb. 11 zeigt die überlagerte Einpassung des Modells um die Genauigkeit der Pose von mehreren Richtungen anzuzeigen.

## Greifen transparenter Objekte

**Figure 12:**
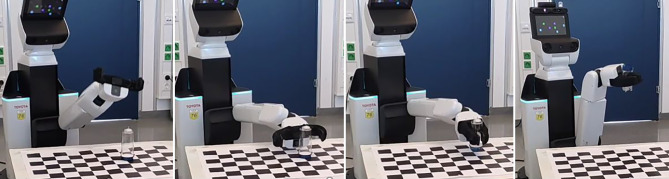

Die vorgestellte Methode um mittels COPE transparente Objekte zu erkennen und deren Pose zu bestimmen, danach die Pose zu verifizieren und zu verbessern, wurde mit einem Toyota HSR Roboter ausgetestet [21]. Abb. 12 zeigt ein Beispiel.

## Schlussfolgerungen

Der Artikel präsentiert Methoden um transparente Objekte wie Kanister, Flaschen und Schläuche in Bildern zu erkennen und die exakte Pose zu bestimmen. Dies ist für viele medizinische und pharmazeutische Anwendungen notwendig, da transparente Objekte allgegenwärtig sind.

Wir zeigen, dass transparente Objekte sehr gut aus Farbbildern gelernt werden könnnen mit Ansätzen wie COPE [14]. Um sowohl die Erkennung zu verifizieren als auch die Pose des Objektes zu verfeinern, verwenden wir einen Ansatz mit Hypothesenbildung und anschließender Verifizierung [7, 8]. Das Ergebnis ist eine Bestätigung, dass das korrekte Objekt erkannt wurde als auch die Pose plausibel und richtig ist.

Dieser Ansatz ist ein wichtiger Schritt um Robotersysteme zu schaffen, die in der Lage sind, zu verstehen, was sie wahrnehmen und was sie tun, um sicherzustellen, dass jede Manipulation eines Objektes mit dem Roboter verifiziert ist und somit den Anforderungen einer regulierten Umgebung entspricht, wie sie in medizinischen und pharmazeutischen Anwendungen der Normalfall sind.

## Footnotes

1 Erstellt für die Sterilitätstests im TraceBot Project https://www.tracebot.eu/.

## References

[1] Biermann, F., Mathews, J., Nießing, B., König, N., Schmitt, R.H.: Automating laboratory processes by connecting biotech and robotic devices—an overview of the current challenges, existing solutions and ongoing developments. Processes **9**(6), 966 (2021). 10.3390/pr9060966

[2] Charoo, N.A., Khan, M.A., Rahman, Z.: Data integrity issues in pharmaceutical industry: Common observations, challenges and mitigations strategies. International Journal of Pharmaceutics **631**, 122503 (2023). 10.1016/j.ijpharm.2022.122503 10.1016/j.ijpharm.2022.122503 36529357

[3] Vagaš, M., Putala, J.: Verification of designed assembly process at research robotized workplace. Applied Mechanics and Materials **844**, 38–43 (2016). 10.4028/www.scientific.net/amm.844.38

[4] Rathmair, M., Haspl, T., Komenda, T., Reiterer, B., Hofbaur, M.: A formal verification approach for robotic workflows. In: 2021 IEEE 20th International Conference on Advanced Robotics (ICAR), pp. 670–675 (2021). 10.1109/icar53236.2021.9659366

[5] Rojas, J., Harada, K., Onda, H., Yamanobe, N., Yoshida, E., Nagata, K., Kawai, Y.: Probabilistic state verification for snap assemblies using the relative-change-based hierarchical taxonomy. In: 2012 12th IEEE-RAS International Conference on Humanoid Robots (Humanoids 2012) (2012). 10.1109/humanoids.2012.6651505

[6] Choi, C., Rus, D.: Probabilistic visual verification for robotic assembly manipulation. In: 2016 IEEE International Conference on Robotics and Automation (ICRA) (2016). 10.1109/icra.2016.7487786

[7] Bauer, D., Patten, T., Vincze, M.: ReAgent: Point cloud registration using imitation and reinforcement learning. In: IEEE/CVF Conference on Computer Vision and Pattern Recognition (CVPR) (2021). 10.1109/cvpr46437.2021.01435

[8] Bauer, D., Patten, T., Vincze, M.: SporeAgent: Reinforced scene-level plausibility for object pose refinement. In: 2022 IEEE/CVF Winter Conference on Applications of Computer Vision (WACV) (2022). 10.1109/wacv51458.2022.00027

[9] Chen, X., Zhang, H., Yu, Z., Opipari, A., Chadwicke Jenkins, O.: Clearpose: Large-scale transparent object dataset and benchmark. In: Avidan, S., Brostow, G., Cissé, M., Farinella, G.M., Hassner, T. (eds.) Computer Vision – ECCV 2022, pp. 381–396. Springer, Cham (2022). 10.1007/978-3-031-20074-8_22

[10] Goodfellow, I., Bengio, Y., Courville, A.: Deep Learning, (2016). MIT Press. http://www.deeplearningbook.org

[11] Patten, T., Park, K., Vincze, M.: DGCM-net: Dense geometrical correspondence matching network for incremental experience-based robotic grasping. Frontiers in Robotics and AI **7** (2020). 10.3389/frobt.2020.00120 10.3389/frobt.2020.00120 PMC7805634 33501286

[12] Thalhammer, S., Leitner, M., Patten, T., Vincze, M.: PyraPose: Feature pyramids for fast and accurate object pose estimation under domain shift. In: 2021 IEEE International Conference on Robotics and Automation (ICRA) (2021). 10.1109/icra48506.2021.9562108

[13] Bauer, D., Patten, T., Vincze, M.: VeREFINE: Integrating object pose verification with physics-guided iterative refinement. IEEE Robotics and Automation Letters **5**(3), 4289–4296 (2020). 10.1109/lra.2020.2996059

[14] Thalhammer, S., Patten, T., Vincze, M.: Cope: End-to-end trainable constant runtime object pose estimation. In: 2023 IEEE/CVF Winter Conference on Applications of Computer Vision (WACV), pp. 2860–2870 (2023). 10.1109/WACV56688.2023.00288

[15] Caron, M., Touvron, H., Misra, I., J’egou, H., Mairal, J., Bojanowski, P., Joulin, A.: Emerging properties in self-supervised vision transformers, pp. 9630–9640 (2021). 10.1109/ICCV48922.2021.00951

[16] Thalhammer, S., Weibel, J.-B., Vincze, M., Garcia-Rodriguez, J.: Self-supervised vision transformers for 3d pose estimation of novel objects. arXiv preprint arXiv:2306.00129 (2023)

[17] Sajjan, S., Moore, M., Pan, M., Nagaraja, G., Lee, J., Zeng, A., Song, S.: Clear grasp: 3d shape estimation of transparent objects for manipulation. In: 2020 IEEE International Conference on Robotics and Automation (ICRA), pp. 3634–3642 (2020). 10.1109/ICRA40945.2020.9197518

[18] Liu, X., Jonschkowski, R., Angelova, A., Konolige, K.: Keypose: Multi-view 3d labeling and keypoint estimation for transparent objects. In: 2020 IEEE/CVF Conference on Computer Vision and Pattern Recognition (CVPR), pp. 11599–11607 (2020). 10.1109/CVPR42600.2020.01162

[19] Xu, H.: TODD dataset. Borealis (2021). 10.5683/SP3/ZJJAJ3

[20] Suchi, M., Neuberger, B., Salykov, A., Weibel, J.-B., Patten, T., Vincze, M.: 3d-dat: 3d-dataset annotation toolkit for robotic vision. In: 2023 IEEE International Conference on Robotics and Automation (ICRA), pp. 9162–9168 (2023). 10.1109/ICRA48891.2023.10160669

[21] Gupta, H., Thalhammer, S., Leitner, M., Vincze, M.: Grasping the inconspicuous. arXiv preprint arXiv:2211.08182 (2022)

